# A longitudinal assessment of trial protocols approved by research ethics committees: The Adherance to SPIrit REcommendations in the UK (ASPIRE-UK) study

**DOI:** 10.1186/s13063-022-06516-1

**Published:** 2022-07-27

**Authors:** Benjamin Speich, Ayodele Odutayo, Nicholas Peckham, Alexander Ooms, Jamie R. Stokes, Ramon Saccilotto, Dmitry Gryaznov, Belinda von Niederhäusern, Bethan Copsey, Douglas G. Altman, Matthias Briel, Sally Hopewell

**Affiliations:** 1 Centre for Statistics in Medicine, Nuffield Department of Orthopaedics, Rheumatology and Musculoskeletal Sciences, University of Oxford, Oxford, UK; 2 Meta-Research Centre, Department of Clinical Research, University Hospital Basel, University of Basel, Basel, Switzerland; 3 Applied Health Research Centre, Li Ka Shing Knowledge Institute of St Michael’s Hospital, Toronto, Ontario Canada; 4 Oxford Clinical Trials Research Unit, NDORMS, University of Oxford, Oxford, UK; 5 Clinical Trial Unit, Department of Clinical Research, University of Basel and University Hospital Basel, Basel, Switzerland; 6 Roche Pharma AG, Grenzach-Wyhlen, Germany; 7 Department of Health Research Methods, Evidence, and Impact, McMaster University, Hamilton, Canada

**Keywords:** Randomised controlled trial, Reporting quality, Trial protocol, Meta-research

## Abstract

**Background:**

To assess the quality of reporting of RCT protocols approved by UK research ethics committees before and after the publication of the Standard Protocol Items: Recommendations for Interventional Trials (SPIRIT) guideline.

**Methods:**

We had access to RCT study protocols that received ethical approval in the UK in 2012 (*n*=103) and 2016 (*n*=108). From those, we assessed the adherence to the 33 SPIRIT items (i.e. a total of 64 components of the 33 SPIRIT items). We descriptively analysed the adherence to SPIRIT guidelines as proportion of adequately reported items (median and interquartile range [IQR]) and stratified the results by year of approval and sponsor.

**Results:**

The proportion of reported SPIRIT items increased from a median of 64.9% (IQR, 57.6–69.2%) in 2012 to a median of 72.5% (IQR, 65.3–78.3%) in 2016. Industry-sponsored RCTs reported more SPIRIT items in 2012 (median 67.4%; IQR, 64.1–69.4%) compared to non-industry-sponsored trials (median 59.8%; IQR, 46.5–67.7%). This gap between industry- and non-industry-sponsored trials increased in 2016 (industry-sponsored: median 75.6%; IQR, 71.2–79.0% vs non-industry-sponsored: median 65.3%; IQR, 51.6–76.3%).

**Conclusions:**

The adherence to SPIRIT guidelines has improved in the UK from 2012 to 2016 but remains on a modest level, especially for non-industry-sponsored RCTs.

**Supplementary Information:**

The online version contains supplementary material available at 10.1186/s13063-022-06516-1.

## Background

Randomised controlled trials (RCTs) provide the highest level of evidence when assessing the potential benefits and potential harms of health care interventions [1, 2]. As the conduct of RCTs is complex, a study protocol describing the essential steps and justification for the trial is needed for efficient and successful completion of a trial [3, 4]. Protocols are not only crucial so that the involved study team has clear guidance about the exact study process; they are also necessary so that external parties, such as funding agencies, research ethics committees, regulatory agencies, medical journals, and systematic reviewers can evaluate the conduct of the study [5]. A meta-research study conducted by Getz and colleagues found that by putting greater attention to study protocols, approximately a third of all protocol amendments submitted to French research ethics committees could have been avoided [6, 7]. Protocols should also clearly define how data is analysed to avoid selective reporting of analyses and outcomes [8]. To improve reporting of important aspects of a trial in study protocols (e.g. treatment allocation, sample size calculation, outcomes, access to final data, or the role of the sponsor [9–15]), an international group of stakeholders founded the SPIRIT (Standard Protocol Items: Recommendations for Interventional Trials) initiative [5, 16]. Pursuing the goal of improving the reporting, the initiative published the SPIRIT recommendations in 2013, a checklist including 33 items that should be transparently reported in trial protocols [5, 16].

To our knowledge, there is no large-scale project assessing the adherence to the SPIRIT reporting guidelines. Hence, we initiated the Adherance to SPIrit Recommendations (ASPIRE) study [17] to evaluate the adherence in reporting SPIRIT items in study protocols that were approved by research ethics committees in Switzerland, the UK, Canada and Germany in 2012 and 2016 (i.e. before, and after the publication of the SPIRIT guidelines). Due to different timelines, as indicated in our protocol [17], the analyses of the ASPIRE sample from Switzerland, Canada, and Germany (ASPIRE-SCAGE) were published earlier [18]. In this study, we assess now if there was an improvement in adherence to SPIRIT guidelines in RCT protocols from the UK (ASPIRE-UK).

## Methods

The international ASPIRE project aims to assess in a repeated cross-sectional design the completeness of RCT protocols before and after the publication of the SPIRIT reporting guidelines. The detailed methods used for this study [17] and the results from ASPIRE-SCAGE were reported previously [18]; here, we report the results for ASPIRE-UK. Additional ASPIRE sub-studies evaluated the fate or RCTs in terms of registration, discontinuation, and non-publication [19], the completeness of reporting in protocols of regulated and non-regulated interventions [20], the use of routinely collected data and patient-reported outcomes, as well as the planning of sub-group analysis in study protocols [17].

### Selection of included protocols of randomised controlled trials

After signing a confidentiality agreement the UK Health Research Authority and Bristol regional research ethics office, which is responsible for 19 research ethics committees, granted us access to clinical trial protocols that were approved in 2012 and 2016 for this study. We included studies that evaluated the effect of an intervention on clinical outcome measures in the frame of a RCT. As units of randomisation, we considered different individuals, groups (cluster-trials), or within individuals (split-body design). We excluded phase 1 trials, feasibility and pilot studies, studies to which we had no access to the full trial protocol, protocols describing more than one RCT, and studies evaluating primarily health economics. Available study protocols were checked for eligibility by one author (2012: AyodeleO; 2016: BS). In 2016 more RCT protocols were available than in 2012; hence, a random sample of protocols was included in 2016 to match the sample size of 2012. We selected the years 2012 and 2016 for our assessment because 2012 should be representative for the completeness of reporting in the study protocol 1 year before SPIRIT was published, while 2016 was chosen to provide sufficient time so that stakeholders (e.g. researchers, funders, ethical committees) can get familiar with the new reporting guideline.

### Data extraction

The SPIRIT checklist includes 33 items that should be reported [5, 16]. To operationalise the assessment of the items this list can be further divided into different components that should be reported to fulfil the item. The 33 items from the SPIRIT checklist can be divided into a total of 270 components. To operationalise the checklist for data extraction the ASPIRE-project team (AyodeleO; BN; SH; MB and DGA) selected the 64 components that were deemed most crucial (at least one from each of the 33 items) that should be reported (see Appendix 1 for selection of 64 components and rational) [17]. We extracted the following information from study protocols: Target sample size, sponsorship (industry- vs non-industry-sponsored), number of trial arms, number of study centres, number of countries where the trial was conducted, hypothesis (superiority vs non-inferiority), and the adherence to the 64 components of the 33 items from the SPIRIT checklist. The data extraction was piloted by several members of the ASPIRE team and everyone involved underwent a calibration process. Data extraction for ASPIRE-UK was conducted by one person (2012: AyodeleO, BC; 2016: BS) and 30% of 2012 and 2016 protocols were double extracted by a second reviewer (2012: BS; 2016: NP, AlexO, JRS). Disagreements were resolved by discussion. Agreement between the data extraction performed by the data extractors was generally high with a median of 6% (interquartile ranges [IQR]: 5–11%) of items needing to be changed due to the second extractor (2012: 8%; IQR: 4–11%; 2016: 6%; IRQ: 5–9%).

### Analysis

We descriptively assessed the number and the proportion of adequately reported SPIRIT items (as median and IQR) in trial protocols approved in 2012 and 2016. The main approach, as defined in our design paper, allowed “for partial credit of individually met subitems or components of major SPIRIT items” [17]. If there was, for example, an item with four components that were included in our extraction, each fulfilled component scored 0.25 points. Sensitivity analyses were conducted for the other two pre-specified approaches rating an item only if all components were met, or rating each component one point (see design paper for further details [17]), and for assigning no points to not applicable items (vs. giving points when the item was not applicable). Analyses were stratified by year of study approval (2012 vs 2016) and by sponsor (industry- vs non-industry-sponsored).

In three separate analyses, we highlight (i) selected SPIRIT item components that achieved an absolute increase in reporting from 2012 to 2016 above 20%, (ii) absolute increase in reporting from selected SPIRIT item components from 2012 to 2016 for components that achieved low reporting (i.e. ≤50%) in 2012, and (iii) selected SPIRIT components for which the reporting was still poor (<60%) in 2016. We conducted beta regression analyses to assess if year of protocol approval, type of sponsor, sample size (in increments of 1000), or multicentre trials were associated with higher adherence to SPIRIT guidelines. All analyses were conducted using STATA 16.1 [21].

## Results

**Fig. 1:**
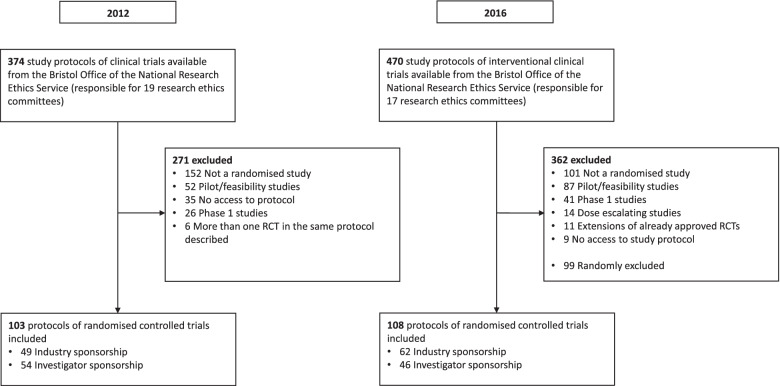
Flow diagram. Abbreviations: RCT, randomised controlled trial

We included 211 study protocols of approved RCTs (103 in 2012, 108 in 2016; Fig. 1). The included trials had a median planned sample size of 200 (IQR 90–400) and approximately half were industry-sponsored trials (52.6%; 111/211). Overall, the majority of RCT protocols were 2-arm studies (74.9%; 158/211). Most protocols were of superiority trials (84.8%; 179/211), and there were more protocols for studies conducted at multiple study centres (76.3%; 161/211; Table 1).

**Table 1:** Baseline characteristics of study protocols from randomised controlled trials that were ethically approved in 2012 or 2016

	2012 (*n*=103)	2016 (*n*=108)	Total (*n*=211)
Target sample size, median (IQR)	180 (90-430)	222 (93-391)	200 (90-400)
Sponsorship			
Industry-sponsored	49 (47.6%)	62 (57.4%)	111 (52.6%)
Non-industry-sponsored	54 (52.4%)	46 (42.6%)	100 (47.4%)
Number of trial arms			
2 arms	76 (73.8%)	82 (75.9)	158 (74.9%)
3 arms	17 (16.5%)	16 (14.8%)	33 (15.6%)
4 arms	7 (6.8%)	7 (6.5%)	14 (6.6%)
>4 arms	3 (2.9%)	3 (2.8%)	6 (2.8%)
Centres			
Single-centre	25 (24.3%)	22 (20.4%)	47 (22.3%)
Multicentre	77 (74.8%)	84 (77.8%)	161 (76.3%)
Unclear	1 (1.0%)	2 (1.9%)	3 (1.4%)
Countries			
National	61 (59.2%)	45 (41.7%)	106 (50.2%)
International	42 (40.8%)	63 (58.3%)	105 (48.8%)
Hypothesis			
Superiority	84 (81.6%)	95 (88.0%)	179 (84.8%)
Non-inferiority	11 (10.7%)	8 (7.4%)	19 (9.0%)
Superiority and non-inferiority	2 (1.9%)	0 (0.0%)	2 (1.0%)
Unclear/not labelled in this regard	6 (5.8%)	5 (4.6%)	11 (5.2%)

*Abbreviations*: *IQR* interquartile range

**Fig. 2:**
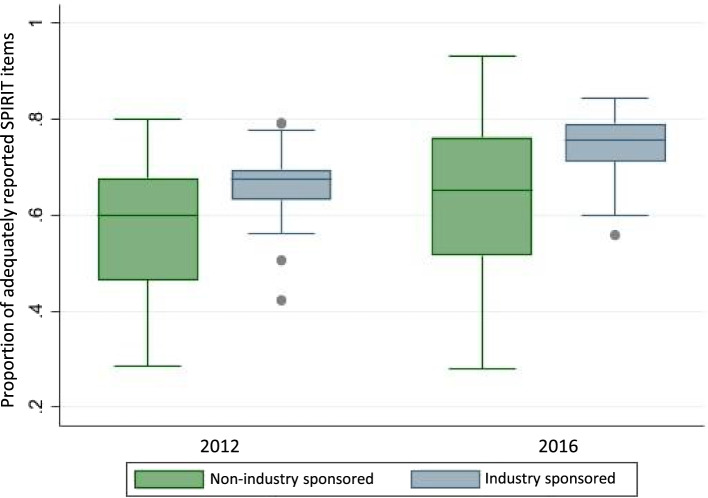
Box-plots of proportion of adequately reported SPIRIT items by year of ethical approval (2012 vs 2016) and sponsor (industry- vs non-industry-sponsored)

The proportion of adequately reported SPIRIT items increased from 64.9% (IQR: 57.6–64.9%) in 2012 to 72.5% (IQR: 65.3–78.3%) in 2016 (Table 2). This translates to a median of 21.4 (IQR: 19.0–22.8), and 23.9 (IQR: 21.6–25.8) correctly reported SPIRIT items in 2012 and 2016, respectively. The proportion of adherence to reporting guidelines was higher in 2012 for industry-sponsored trials (67.4%; IQR 64.1–69.4%) compared to non-industry-sponsored RCTs (59.8%; IQR: 46.5–67.7%). This difference was even larger in 2016 (industry-sponsored: 75.6%; IQR 71.2–79.0%; non-industry-sponsored: 65.3%; IQR: 51.6–76.3%; Fig. 2). All the conducted sensitivity analyses confirmed that the reporting improved in 2016 and was in general better in industry-sponsored RCTs (see supplementary Table S1, appendix).

**Table 2:** Adherence to SPIRIT (Standard Protocol Items: Recommendations for Interventional Trials) reporting guidelines in randomised clinical trials that received ethical Approval in 2012 and 2016, respectively

	2012			2016		
	Industry sponsorship (***n***=49)	Non-industry sponsorship (***n***=54)	Total 2012 (***n***=103)	Industry sponsorship (***n***=62)	Non-industry sponsorship (***n***=46)	Total 2016 (***n***=108)
SPIRIT items (*n*=33) adequately reported (median; IQR)^a^	22.5 (20.8–22.9)	19.8 (15.3–22.3)	21.4 (19.0–22.8)	25.0 (23.5–26.1)	21.5 (17.0–25.2)	23.9 (21.6–25.8)
Proportion of SPIRIT items adequately reported (median; IQR)	67.4% (64.1–69.4%)	59.8% (46.5–67.7%)	64.9% (57.6–69.2%)	75.6% (71.2–79.0%)	65.3% (51.6–76.3%)	72.5% (65.3–78.3%)

^a^Using the “major item approach” in which adequately reported components of items receive partial credit. See design paper for more details about individual approach [17] and appendix Table S1 for sensitivity analyses using other pre-defined approaches

The reporting for all 33 SPIRIT items, consisting of the 64 extracted components, are reported in Table S2 (Appendix). A total of 12 selected SPIRIT item components had an absolute increase in adequate reporting above 20% (Table 3). The highest increase was seen for components belonging to item 28 “Declaration of Interests” (absolute increase: 53.1%; 2012: 11.7%; 2016: 64.8%), item 30 “Ancillary and post-trial care” (absolute increase: 43.9%; 2012: 11.7%; 2016: 55.6%), and item 19 “Data entry and coding” (absolute increase: 45.2%; 2012: 18.7%; 2016: 63.9%). A total of 21 selected SPIRIT item components had relatively low reporting in 2012 (i.e. ≤50%), with some items showing no indication for improvement (i.e. “Name of protocol contributors/authors, “Eligibility criteria for study centres and who will perform the intervention”, “Location of participant recruitment”, “Person who will enrol/assign participants”; Table S3). In 2016, we still identified an overall adherence to reporting guidelines in 2016 below 60% for 18 out of the 64 selected SPIRIT item components (Table 4). The components that were least often reported belonged to item 31c “Plans for granting access to full trial protocol” (7.4%; 8/108), item 5a “Names of protocol contributors/authors” (11.1%; 12/108), and item 29 “Who will have access to full dataset” (23.2%, 25/108). Conducting a multivariable beta regression showed that the following variables were associated with higher completeness of reporting: ethical approval in 2016 (vs 2012), industry sponsorship (vs non-industry sponsorship), and multicentre RCTs (vs. single centre, Table S4).

**Table 3:** Selected SPIRIT item components with highest absolute improvement in adequate reporting (2016 vs 2012)

SPIRIT component	Belonging to SPIRIT Item Number	2012			2016			Absolute increase
		Industry sponsorship (***n***=49)	Non-industry sponsorship (***n***=54)	Total 2012 (***n***=103)	Industry sponsorship (***n***=62)	Non-industry sponsorship (***n***=46)	Total 2016 (***n***=108)	
Basic study design in title	1	31 (63.3%)	9 (16.7%)	40 (38.8%)	53 (85.5%)	24 (52.2%)	77 (71.3%)	32.5%
Name and contact details of sponsor	5b	12 (24.5%)	16 (29.6%)	28 (27.2%)	31 (50.0%)	31 (67.4%)	62 (57.4%)	30.2%
Steering Committee General Membership and Role	5d	35 (71.4%)	45 (83.3%)	81 (77.7%)	56 (90.3%)	39 (84.8%)	73 (98.0%)	20.3%
Of which not applicable		30 (61.2%)	29 (53.7%)	59 (57.3%)	46 (74.2%)	24 (52.2%)	70 (64.8%)	
Research question described and justified	6a	11 (22.5%)	9 (16.7%) 2NA	20 (19.4%)	35 (56.5%)	13 (28.3%)	48 (44.4%)	25.0%
Strategies to improve or monitoring of adherence	11c	33 (67.4%)	34 (63.0%)	67 (65.1%)	57 (91.9%)	40 (87.0%)	97 (89.8%)	24.7%
Of which not applicable		9 (18.4%)	25 (46.3%)	34 (33.0%)	23 (37.1%)	28 (60.9%)	51 (41.2%)	
Expected recruitment rate	15	7 (14.3%)	15 (14.3%)	22 (21.4%)	20 (32.3%)	25 (45.4%)	45 (41.7%)	20.3%
Method for generation of random sequence	16a	21 (42.8%)	29 (53.7%)	50 (48.5%)	51 (82.3%)	30 (65.2%)	81 (75.0%)	26.5%
Strategies to promote participant retention and complete follow-up	18b	9 (18.4%)	20 (38.0%)	29 (28.4%)	38 (61.3%)	16 (34.8%)	54 (50.0%)	21.6%
Of which not applicable		2 (4.1%)	2 (3.7%)	4 (3.9%)	-	-	-	
Data entry and coding	19	11 (22.5%)	9 (15.5%)	20 (18.7%)	49 (79.0%)	20 (43.5%)	69 (63.9%)	45.2%
Process for making amendments described	25	27 (55.1%)	8 (14.8%)	35 (34.0%)	50 (80.7%)	21 (45.7%)	71 (65.7%)	31.7%
Declaration of Interests	28	8 (16.3%)	4 (7.4%)	12 (11.7%)	60 (96.8%)	10 (21.7%)	70 (64.8%)	53.1%
Ancillary and post-trial care	30	4 (8.2%)	8 (14.8%)	12 (11.7%)	29 (46.8%)	31 (67.4%)	60 (55.6%)	43.9%

**Table 4:** Selected SPIRIT item components which were least often adequately reported (<60%) in study protocols of randomised controlled trial protocols that were approved in 2016

SPIRIT component	Belonging to SPIRIT Item Number	Industry sponsorship (***n***=62)	Non-industry sponsorship (***n***=46)	Total 2016 (***n***=108)
Names of protocol contributors/authors	5a	5 (8.1%)	7 (15.2%)	12 (11.1%)
Name and contact details of sponsor	5b	31 (50.0%)	31 (67.4%)	62 (57.4%)
Research question described and justified	6a	35 (56.5%)	13 (28.3%)	48 (44.4%)
Countries where data will be collected listed	9	9 (14.5%)	36 (78.3%)	45 (41.7%)
Eligibility criteria for study centres and who will perform the intervention	10	8 (12.9%)	24 (52.2%)	32 (29.6%)
Sample size: assumed values for outcome	14	41 (36.9%)	20 (43.5%)	61 (56.5%)
Location of participant recruitment	15	7 (11.3%)	36 (78.3%)	43 (39.8%)
Person(s) who will recruit participants	15	5 (8.1%)	31 (61.4%)	36 (33.3%)
Expected recruitment rate	15	20 (32.3%)	25 (45.4%)	45 (41.7%)
Person who will enrol/assign participants	16c	12 (19.4%)	17 (37.0%)	29 (26.7%)
Personnel who will collect data	18a	19 (30.7%)	24 (52.2%)	43 (39.8%)
Strategies to promote participant retention and complete follow-up	18b	38 (61.3%)	16 (34.8%)	54 (50.0%)
DMC is planned or why it is not planned	21a	41 (66.1%)	22 (47.8%)	63 (58.3%)
Audits/external monitoring described	23	26 (41.9%)	3 (5.5%)	29 (26.9%)
Who will have access to full dataset	29	18 (29.0%)	7 (15.2%)	25 (23.2%)
Plans to disseminate trial results to key stakeholders/publication provided	31a	29 (46.8%)	31 (67.4%)	60 (55.6%)
Authorship eligibility criteria	31b	23 (37.1%)	15 (32.6%)	38 (35.2%)
Plans for granting access to full trial protocol	31c	6 (9.7%)	2 (4.4%)	8 (7.4%)

## Discussion

The results of our study showed that the proportion of reported SPIRIT items in RCT study protocols approved by UK research ethics committees increased between 2012 and 2016. This improvement was most evident in industry-sponsored studies which reached an improvement of more than 10%. Nevertheless, large deficiencies in the reporting of important SPIRIT items remain, given that industry-sponsored studies report 1 out of 4 (24.4%) SPIRIT items inadequately and non-industry-sponsored RCTs miss to report 1 out of 3 items (34.7%).

Our results are in line with an assessment conducted by Kyte and colleagues on a sample of 75 non-industry-sponsored RCTs from the UK National Institute for Health Research Health Technology Assessment that were approved in 2012 and 2013 [22]. They concluded that 63% of SPIRIT items were adequately reported which is comparable to the 60% we identified in non-industry-sponsored RCTs approved in 2012. The part of the ASPIRE study [17] that assessed the completeness of reporting of study protocols in Switzerland, Canada, and Germany (published previously due to different timelines of available resources) found similar adherence to SPIRIT guidelines for industry-sponsored RCTs in 2016 as within the UK sample (ASPIRE-SCAGE: 77% [18]; ASPIRE-UK: 76%). However, it appeared that the reporting in Switzerland, Canada, and Germany had a greater improvement in non-industry-sponsored trials (from 64% in 2012 to 76% in 2016) compared to the sample in the UK (from 60% in 2012 to 65% in 2016). In ASPIRE-SCAGE the strongest improvement in completeness of reporting in non-industry-sponsored RCTs was seen in trials approved by Swiss ethics committees [18]. It is possible that the SPIRIT-based protocol template [23] that was introduced by Swiss ethics in the frame of the new Swiss legislation on human research in 2014, might be related to this improvement (e.g. providing guidance for researchers on how to write a protocol). This approach was also implemented by the journal *Trials*, as they provide a template following closely all SPIRIT items, aiming to make protocols more structured [24]. The Health Research Authority from the UK has also provided an optional template in 2016 [25]. An additional assessment of completeness of reporting in study protocols (e.g. from 2020) is needed to assess if this intervention has improved adherence to the SPIRIT Statement. In order that well-reported study protocols are useful to others (e.g. other researchers, patients, funding bodies), they should also be publicly available. In our sample, only 6% of protocols clarified who will have access to the final study protocol. Hence, it is important that not only the completeness of reporting of study protocols is improved, but also the public sharing of protocols is actively promoted [26] (or even enforced in parallel by journals publishing the results). Other important aspects to increase transparency include increasing the rate of registered trials and published trials which we have assessed in the frame of the ASPIRE project [17] and published in a separate sub-study [19, 27].

The following limitations should be noted: First, given the high workload of extracting all 64 components from ethically approved study protocols and due to limited resources, only approximately 30% of studies were extracted in duplicate. In addition, study protocols from 2012 (AO) and 2016 (BS) were extracted separately by different main extractors. We tried to control for this limitation by conducting pilot assessments and conducting calibration extractions so that extractors use the same judgement when assessing the SPIRIT items. The agreement between data extractors was relatively high with only around 6% of items needed to be revised after double extraction. However, we cannot fully exclude that this limitation might have influenced our study results. Second, some of the SPIRIT items assessed were always by default fulfilled as the RCT received already ethical approval (i.e. items “research ethical approval”, “consent form provided”). We do not believe that this could have influenced the overall study results. Third, the SPIRIT checklist is a guidance for writing, supporting authors to implement the most essential information in a manuscript. It was, however, not developed as a measurement tool to assess reporting quality [28]. As the SPIRIT checklist includes 33 items consisting of 270 components we had to operationalise the checklist to be able to extract data in a meaningful way (see study protocol and for more details [17] and Appendix 1 for selection of 64 components). Even though we conducted different sensitivity analyses how to credit individual components (supplementary Table S1, appendix), as pre-defined in our study protocol [17], showing all the same overall result, we cannot completely exclude the possibility that selecting different components, to operationalise the assessment of complete reporting, might have influenced our results. Fourth, our study cannot clarify if the improvement in reporting is due to the publication of the SPIRIT guidelines. Other factors, such as the awareness of the importance of study protocols and transparent reporting in general, as well as better knowledge and knowledge transfer in RCT methodology amongst clinical scientists, might have also influenced the study result.

In conclusion, adherence to SPIRIT guidelines has improved in the UK from 2012 to 2016 but remains on a modest level, especially for non-industry-sponsored RCTs. Protocol templates closely aligning with the SPIRIT guidelines might be a way to further improve the reporting in trial protocols.

## Supplementary Information

**Additional file 1: Appendix 1.** The 64 components that were extracted to assess adherence of the 33 SPIRIT items (as defined for the ASPIRE project (1)). **Table S1.** Sensitivity analysis using different approaches* to assess the adherence to SPIRIT guidelines. **Table S2.** Adherence to individual SPIRIT items stratified by year and sponsorship. **Table S3.** Absolute increase in adequate reported selected SPIRIT item components (2012 vs 2016) for components that were not commonly reported (i.e. ≤50%) in 2012. **Table S4.** Results from beta regression to assess what characteristics are associated with higher proportion of adequate reporting.

## Data Availability

The collaborating research ethic committee granted us access to approved study protocols under the condition that only aggregated data will be published. Hence, no individual data will be shared for this project.
