# Supplementary material for: A longitudinal assessment of trial protocols approved by research ethics committees: The Adherance to SPIrit REcommendations in the UK (ASPIRE-UK) study
Source: Trials. 2022 Jul 27;23:601. doi: 10.1186/s13063-022-06516-1 (PMC9327179; doi:10.1186/s13063-022-06516-1)
Supplement: Supplementary file 1 — Additional file 1: Appendix 1. The 64 components that were extracted to assess adherence of the 33 SPIRIT items (as defined for the ASPIRE project (1)). Table S1. Sensitivity analysis using different approaches* to assess the adherence to SPIRIT guidelines. Table S2. Adherence to individual SPIRIT items stratified by year and sponsorship. Table S3. Absolute increase in adequate reported selected SPIRIT item components (2012 vs 2016) for components that were not commonly reported (i.e. ≤50%) in 2012. Table S4. Results from beta regression to assess what characteristics are associated with higher proportion of adequate reporting. [file 13063_2022_6516_MOESM1_ESM.docx]

**Appendix**

**A longitudinal assessment of the reporting quality of ethically approved trial protocols: The Adherance to SPIrit Recommendations in the UK (ASPIRE-UK) study**

Benjamin Speich, Ayodele Odutayo, Nicholas Peckham, Alexander Ooms, Jamie R. Stokes, Ramon Saccilotto, Dmitry Gryaznov, Belinda von Niederhäusern, Bethan Copsey, Douglas G Altman, Matthias Briel, Sally Hopewell

**Appendix 1:** The 64 components that were extracted to assess adherence of the 33 SPIRIT items (as defined for the ASPIRE project (1)).

The 64 components were selected as followed as indicated in our study protocol (Gryaznov et al., 2020, *BMJ Open*; (1)): “*The complete SPIRIT checklist includes 270 individual components grouped under 33 separate headings. For instance, the heading “sample size calculation” has nine components which are relevant to the calculation of sample size, including but not limited to the statistical test used to calculate the sample size, type I error, type II error, and the minimum anticipated difference or event rate. For our assessment, we will consider all 33 major items or subitems indicated by letters (e.g. 18a, 18b) of the SPIRIT checklist. However, we need to rationalize the checklist for data extraction purposes, i.e. some of the individual components of SPIRIT items or subitems will not be considered, because we feel that such a level of detail is not helpful for our empirical analysis and adds unnecessary complexity and burden for data extractors. The process of identifying which components to include in data extractions was as follows. First, all items and components were included where the heading related to the formulation of a research question using the PICO structure (Population, Intervention, Comparator and Outcome). These were headings that defined the target population, defined the intervention used and any comparators, and defined the outcomes of the study. These headings were considered important because they are relevant to defining the research question of interest for each clinical trial. Likewise, all SPIRIT items were included for headings related to sample size calculation, random sequence generation, allocation concealment, and blinding. These headings were chosen because of their importance for reducing bias in clinical trials. For the remaining SPIRIT headings, two investigators (Ayodele Odutay and Belinda von Niederhäusern) independently reviewed each of the items and components under the heading and selected components that encompassed the core message of the heading. These selections were compared and reviewed with three additional collaborators (Prof. Sally Hopewell, Prof Matthias Briel, and Prof. Douglas G. Altman) to achieve consensus on the final selection*.”

| **Component to assess SPIRIT items** | **Options for the answer** |
| --- | --- |
| 1. Title: Basic study design, patient population, and intervention provided in study title (if applicable trial acronym)? (reporting) | Yes |
|  | No |
| 2. Trial Registration: Registry name and trial identifier provided? (reporting) | Yes |
|  | No |
| 3. Protocol: Version Number and date provided? (reporting) | Yes |
|  | No |
| 4. Funding: Sources of financial and non-financial support declared? (reporting) | Yes |
|  | No |
| 5. Roles and Responsibilities: Names of protocol contributors/ authors provided? (reporting) | Yes |
|  | No |
| 6. Roles and Responsibilities: Name and contact details of sponsor provided? (reporting) | Yes |
|  | No |
| 7. Roles and Responsibilities: Role of sponsor and funder in trial described? (reporting) | Yes |
|  | No |
| 8. Roles and Responsibilities: Steering Committee General Membership and Role described? (reporting) | Yes |
|  | No |
|  | Not applicable |
| 9. Background and rationale: Is research question described and justified? (as a minimum, we expect a systematic search, see info) (reporting) | Yes |
|  | No |
| 10. Background and rationale: Comparator choice explained? (reporting) | Yes |
|  | No |
| 11. Objectives: Specific objectives described for each comparison (if multiple)? (reporting) | Yes |
|  | No |
| 12. Trial design: Trial design described? (trial type (eg, parallel group, crossover, factorial, single group), allocation ratio, and framework (eg, superiority, equivalence, noninferiority, exploratory)) (reporting) | Yes |
|  | No |
| 13. Study Setting: Are countries where data will be collected listed? (reporting) | Yes |
|  | No |
| 14. Eligibility criteria: Inclusion and exclusion criteria for trial participants described? (reporting) | Yes |
|  | No |
| 15. Eligibility criteria: Inclusion and exclusion criteria for study centres and individuals who will perform the intervention described? (reporting) | Yes |
|  | No |
|  | Not applicable |
| 16. Intervention(drug): Generic Name, Dose and Schedule of intervention described? (reporting) | Yes |
|  | No |
|  | Not applicable |
| 17. Intervention(non-drug): Setting of intervention administration described? (reporting) | Yes |
|  | No |
|  | Not applicable |
| 18. Intervention(non-drug): Individuals administering interventions (e.g. expertise) mentioned? (reporting) | Yes |
|  | No |
|  | Not applicable |
| 19. Interventions - Modifications: Standard criteria for modifications of interventions described? (reporting) | Yes |
|  | No |
|  | Not applicable |
| 20. Interventions - Adherence: Are strategies to improve adherence or any procedures for monitoring adherence described? (reporting) | Yes |
|  | No |
|  | Not applicable |
| 21. Interventions - Concomitant care: Permitted care and interventions during trial described? (reporting) | Yes |
|  | No |
| 22. Primary Outcome: Specific measurement variable described? (reporting) | Yes |
|  | No |
|  | Not applicable |
| 23. Primary Outcome: Analysis metric (e.g. change from baseline) described? (reporting) | Yes |
|  | No |
|  | Not applicable |
| 24. Primary Outcomes: Is time point of measurement mentioned? (reporting) | Yes |
|  | No |
|  | Not applicable |
| 25. Participant timeline: Timing of visit for participants described (e.g. schematic diagram)? (reporting) | Yes |
|  | No |
| 26. Sample size: Estimated number total or per group mentioned? (reporting) | Yes |
|  | No |
| 27. Sample size: Outcome used for samples size calculation described? (reporting) | Yes |
|  | No |
|  | Not applicable |
| 28. Sample size: Assumed values for outcome in each study group provided? (reporting) | Yes |
|  | No |
|  | Not applicable |
| 29. Sample size: Alpha value provided? (reporting) | Yes |
|  | No |
|  | Not applicable |
| 30. Sample size: Statistical Power provided? (reporting) | Yes |
|  | No |
|  | Not applicable |
| 31. Sample size: Rationale for intended sample size if not derived statistically provided? (reporting) | Yes |
|  | No |
|  | Not applicable |
| 32. Recruitment: Location of participant recruitment described? (reporting) | Yes |
|  | No |
| 33. Recruitment: Person(s) who will recruit participants described? (reporting) | Yes |
|  | No |
| 34. Recruitment: Expected recruitment rate provided? (reporting) | Yes |
|  | No |
| 35. Allocation: Method for generation of random sequence described? ( e.g. computer-generated random numbers) (reporting) | Yes |
|  | No |
|  | Not applicable |
| 36. Allocation: Allocation concealment mechanism described? (reporting) | Yes |
|  | No |
|  | Not applicable |
| 37. Allocation: Person who will enroll/assign participants described? (reporting) | Yes |
|  | No |
|  | Not applicable |
| 38. Blinding: Status of participants described? (reporting) | Yes |
|  | No |
| 39. Blinding: Status of care providers described? (reporting) | Yes |
|  | No |
| 40. Blinding: Status of outcome assessors described? (reporting) | Yes |
|  | No |
| 41. Blinding: Conditions when unblinding is permissible mentioned? (reporting) | Yes |
|  | No |
|  | Not applicable |
| 42. Data Collection: Personnel who will collect data specified? (reporting) | Yes |
|  | No |
| 43. Data collection: Strategies to promote participant retention and complete follow-up described? (reporting) | Yes |
|  | No |
| 44. Data Management: Data entry and coding processes described? (reporting) | Yes |
|  | No |
| 45. Statistical Methods: Main analysis for primary outcome including analysis methods for statistical comparisons described? (reporting) | Yes |
|  | No |
| 46. Statistical Methods: Definition of subgroup categories provided? (reporting) | Yes |
|  | No |
| 47. Statistical Methods: Does the protocol define which participants will be included in the main analysis in terms of protocol adherence and missing data? (reporting) | Yes |
|  | No |
| 48. Data Monitoring Committee: Is it explicitly reported whether a DMC is planned or why it is not planned? (reporting) | Yes |
|  | No |
| 49. Data Monitoring: Reported who has ultimate authority to stop the trial? (reporting) | Yes |
|  | No |
| 50. Harms: Plans for collecting, assessing, reporting, managing anticipated/unanticipated adverse events provided? (reporting) | Yes |
|  | No |
| 51. Auditing: Procedures of audits and/or external monitoring described (e.g. clinical trial unit/CROs)? (reporting) | Yes |
|  | No |
| 52. Research Ethics Approval: Where approval has been obtained, or plans for seeking approval, provided? (should always be yes in this study) (reporting) | Yes |
|  | No |
| 53. Protocol Amendments: Process for making amendments described? (reporting) | Yes |
|  | No |
| 54. Consent or Assent: Informed Consent process described? (reporting) | Yes |
|  | No |
| 55. Consent or Assent – Ancillary Studies: Process to obtain additional consent for collection and use of data and biological specimens described? (reporting) | Yes |
|  | No |
|  | Not applicable |
| 56. Confidentiality: Described how data will be collected, kept secure, and maintained during the trial? (reporting) | Yes |
|  | No |
| 57. Declaration of Interests: Financial and other competing interests clearly stated? (reporting) | Yes |
|  | No |
| 58. Access to data: Is it clearly mentioned who will have access to full dataset after trial completion? (reporting) | Yes |
|  | No |
| 59. Ancillary and post-trial care: Any plans to provide or pay for ancillary care during the trial provided? (reporting) | Yes |
|  | No |
| 60. Dissemination Policy: Plans to disseminate trial results to key stakeholders/publication provided? (reporting) | Yes |
|  | No |
| 61. Dissemination Policy: Authorship eligibility criteria described? | Yes |
|  | No |
| 62. Dissemination Policy: Plans for granting access to full trial protocol provided? (reporting) | Yes |
|  | No |
| 63. Informed Consent Materials: Model consent and/or assent forms provided (e.g in Appendix)? (reporting) | Yes |
|  | No |
| 64. Biological Specimens: Details of specimen collection provided? (reporting) | Yes |
|  | No |
|  | Not applicable |

**Table S1:** Sensitivity analysis using different approaches* to assess the adherence to SPIRIT guidelines

|  | **2012** | | | **2016** | | |
| --- | --- | --- | --- | --- | --- | --- |
|  | **Industry sponsorship (n=49)** | **Non-industry sponsorship (n=54)** | **Total 2012 (n=103)** | **Industry sponsorship (n=62)** | **Non-industry sponsorship (n=46)** | **Total 2016 (n=108)** |
| **Major item approach (allowing for partial credit) NA=1*** |  |  |  |  |  |  |
| SPIRIT items (n=33) adequately reported (median; IQR) | 22.5  (20.8 – 22.9) | 19.8  (15.3 – 22.3) | 21.4  (19.0 - 22.8) | 25.0  (23.5 – 26.1) | 21.5  (17.0 – 25.2) | 23.9  (21.6 – 25.8) |
| Proportion of SPIRIT items adequately reported (median; IQR) | 67.4%  (64.1 – 69.4%) | 59.8%  (46.5 – 67.7%) | 64.9%  (57.6 – 69.2%) | 75.6%  (71.2 – 79.0%) | 65.3%  (51.6 – 76.3%) | 72.5%  (65.3 – 78.3%) |
| **Major item approach (allowing for partial credit) NA=0*** |  |  |  |  |  |  |
| SPIRIT items (n=33) adequately reported (median; IQR) | 19.7  (18.2-21.0) | 17.2  (12.2-19.9) | 18.9  (16.3-20.3) | 22.7  (21.7-24.5) | 19.0  (14.5-23.0) | 22.1  (19.4-23.9) |
| Proportion SPIRIT items adequately reported (median; IQR) | 59.0%  (55.2-63.5%) | 52.0%  (37.0-60.2%) | 57.2%  (49.4-61.5%) | 68.8%  (65.8-74.1%) | 57.6%  (43.9-69.8%) | 66.9%  (58.6-72.5%) |
| **Major Item approach (simple) NA=1*** |  |  |  |  |  |  |
| SPIRIT items (n=33) adequately reported (median; IQR) | 18.0  (17.0-19.0) | 16.0  (11.0-19.0) | 18.0  (15.0-19.0) | 21.0  (19.0-22.0) | 16.5  (12.0-21.0) | 20.0  (17.0-22.0) |
| Proportion SPIRIT items adequately reported (median; IQR) | 54.5%  (51.5-57.6%) | 48.5%  (33.3-57.6%) | 54.5%  (45.5-57.6%) | 63.6%  (57.6-66.7%) | 50.0%  (36.4-63.6%) | 60.6%  (51.5-66.7%) |
| **Major Item approach (simple) NA=0*** |  |  |  |  |  |  |
| SPIRIT items (n=33) adequately reported (median; IQR) | 17.0  (16.0-18.0) | 15.0  (10.0-18.0) | 17.0  (14.0-18.0) | 20.0  (19.0-22.0) | 16.0  (12.0-20.0) | 19.5  (17.0-22.0) |
| Proportion of SPIRIT items adequately reported (median; IQR) | 51.5%  (48.5-54.5%) | 45.6%  (30.3-54.5%) | 51.5%  (42.4-54.5%) | 60.6%  (57.6-66.7%) | 48.5%  (36.4-60.6%) | 59.1%  (51.5-66.7%) |
| **All item approach NA=1*** |  |  |  |  |  |  |
| SPIRIT item components (n=64) adequately reported (median; IQR) | 43.3  (41.3-45.3) | 39.5  (32.0-44.0) | 42.3  (37.0-45.0) | 46.3  (45.3-48.3) | 43.0  (35.0-49.0) | 46.3  (42.1-49.0) |
| Proportion SPIRIT items adequately reported (median; IQR) | 67.6%  (64.5-70.7%) | 61.7%  (50.0-68.8%) | 66.0%  (57.8-70.3%) | 72.3%  (70.7-75.4%) | 67.2%  (54.7-76.6%) | 72.3%  (65.8-76.6%) |
| **All item approach NA=0*** |  |  |  |  |  |  |
| SPIRIT item components (n=64) adequately reported (median; IQR) | 36.3  (35.3-39.3) | 33.0  (24.0-38.0) | 32.3  (30.0-39.0) | 40.3  (38.3-43.3) | 35.5  (27.0-43.0) | 39.3  (34.6-43.0) |
| Proportion of SPIRIT items adequately reported (median; IQR) | 56.6%  (55.1-61.3%) | 51.6%  (37.5-59.4%) | 55.1%  (46.9-60.9%) | 62.9%  (59.8-67.6%) | 55.5%  (42.2-67.2%) | 61.3%  (54.1-67.2%) |

*See design-paper for more information about individual approaches (1).

Abbreviations: NA= Not applicable

**Table S2:** Adherence to individual SPIRIT items stratified by year and sponsorship

|  |  | **2012** | | | **2016** | | |
| --- | --- | --- | --- | --- | --- | --- | --- |
| **Variable** | **Spirit Item Number** | **Industry sponsorship (n=49)** | **Non-industry sponsorship (n=54)** | **Total 2012 (n=103)** | **Industry sponsorship (n=62)** | **Non-industry sponsorship (n=46)** | **Total 2016 (n=108)** |
| Basic study design in Title | 1 | 31 (63.3%) | 9 (16.7%) | 40 (38.8%) | 53 (85.5%) | 24 (52.2%) | 77 (71.3%) |
| Trial registration | 2 | 33 (67.4%) | 17 (31.5%) | 50 (48.5%) | 54 (87.1%) | 19 (41.3%) | 73 (67.6%) |
| Protocol version, number and date | 3 | 49 (100.0%) | 48 (88.9%) | 97 (94.2%) | 56 (90.3%) | 45 (97.9%) | 101 (93.5%) |
| Funding sources | 4 | 49 (100.0%) | 34 (63.0%) | 75 (72.8%) | 62 (100.0%) | 36 (78.3%) | 98 (90.7%) |
| Names of protocol contributors/ authors | 5a | 4 (8.2%) | 8 (13.8%) | 12 (11.2%) | 5 (8.1%) | 7 (15.2%) | 12 (11.1%) |
| Name and contact details of sponsor | 5b | 12 (24.5%) | 16 (29.6%) | 28 (27.2%) | 31 (50.0%) | 31 (67.4%) | 62 (57.4%) |
| Role of sponsor and funder in trial | 5c | 49 (100.0%) | 1 (1.9%) | 50 (51.5%) | 61 (98.4%) | 5 (10.9%) | 66 (61.1%) |
| Steering Committee General Membership and Role | 5d | 35 (71.4%) | 45 (83.3%) | 81 (77.7%) | 56 (90.3%) | 39 (84.8%) | 73 (98.0%) |
| Of which Not Applicable |  | 30 (61.2%) | 29 (53.7%) | 59 (57.3%) | 46 (74.2%) | 24 (52.2%) | 70 (64.8%) |
| Research question described and justified | 6a | 11 (22.5%) | 9 (16.7%) 2NA | 20 (19.4%) | 35 (56.5%) | 13 (28.3%) | 48 (44.4%) |
| Comparator choice explained | 6b | 48 (98.0%) | 48 (88.9%) | 96 (93.2%) | 46 (74.2%) | 36 (78.3%) | 82 (75.9%) |
| Specific objectives described | 7 | 45 (91.8%) | 52 (96.3%) | 97 (94.2%) | 59 (95.2%) | 39 (84.8%) | 98 (90.7%) |
| Trial design described | 8 | 46 (93.9%) | 43 (79.6%) | 89 (86.4%) | 59 (95.2%) | 39 (84.8%) | 98 (90.7%) |
| Countries where data will be collected listed | 9 | 14 (28.6%) | 43 (79.6%) | 57 (55.3%) | 9 (14.5%) | 36 (78.3%) | 45 (41.7%) |
| Eligibility criteria for trial participants | 10 | 49 (100.0%) | 54 (100.0%) | 103 (100.0%) | 62 (100.0%) | 45 (97.8%) | 107 (99.1%) |
| Eligibility criteria for study centres and who will perform the intervention | 10 | 9 (18.4%) | 24 (44.4%) | 33 (32.0%) | 8 (12.9%) | 24 (52.2%) | 32 (29.6%) |
| Of which Not Applicable |  | 6 (12.2%) | 19 (35.2%) | 25 (24.3%) | 3 (4.8%) | 19 (41.3%) | 22 (20.4%) |
| Individuals administering interventions (non-drug) | 10 | 49 (100.0%) | 51 (93.1%) | 100 (97.1) | 62 (100.0%) | 38 (82.6%) | 100 (92.6%) |
| Of which Not Applicable |  | 44 (89.8%) | 18 (33.3%) | 62 (60.2%) | 51 (82.3%) | 13 (28.3%) | 64 (59.3%) |
| Generic Name, Dose and Schedule of intervention | 11a | 48 (98.0%) | 54 (100.0%) | 102 (99.0%) | 60 (96.8%) | 46 (100.0%) | 106 (92.2%) |
| Of which Not Applicable |  | 5 (10.2%) | 36 (66.7%) | 41 (39.8%) | 6 (9.7%) | 32 (69.6%) | 38 (35.2%) |
| Setting of intervention administration | 11a | 49 (100.0%) | 52 (96.3%) | 104 (97.2%) | 62 (100.0%) | 35 (76.1%) | 97 (89.8%) |
| Of which Not Applicable |  | 44 (89.8%) | 18 (33.3%) | 62 (60.2%) | 54 (87.1%) | 12 (26.1%) | 66 (61.1%) |
| Criteria for modifications of interventions | 11b | 33 (67.4%) | 33 (61.1%) | 66 (64.1%) | 50 (80.7%) | 32 (69.6%) | 82 (75.9%) |
| Of which Not Applicable |  | 5 (10.2%) | 16 (29.6%) | 21 (20.4%) | 7 (11.3%) | 17 (37.0%) | 24 (22.2%) |
| Strategies to improve or monitoring of adherence | 11c | 33 (67.4%) | 34 (63.0%) | 67 (65.1%) | 57 (91.9%) | 40 (87.0%) | 97 (89.8%) |
| Of which Not Applicable |  | 9 (18.4%) | 25 (46.3%) | 34 (33.0%) | 23 (37.1%) | 28 (60.9%) | 51 (41.2%) |
| Permitted concomitant care | 11d | 46 (93.9%) | 24 (44.4%) | 70 (68.0%) | 59 (95.2%) | 16 (34.8%) | 75 (69.4%) |
| Primary Outcome: Specific measurement variable | 12 | 49 (100.0%) | 51 (94.4%) | 100 (97.1%) | 57 (91.9%) | 39 (84.8%) | 96 (88.9%) |
| Of which Not Applicable |  | 0 (0.0%) | 4 (7.4%) | 4 (3.9%) | - | - | - |
| Primary Outcome: Analysis metric | 12 | 47 (95.9%) | 39 (72.2%) | 86 (83.5%) | 57 (91.9%) | 33 (71.7%) | 90 (83.2%) |
| Of which Not Applicable |  | 7 (14.3%) | 14 (25.9%) | 21 (20.4%) | 18 (29.0%) | 11 (23.9%) | 29 (26.9%) |
| Primary Outcomes: time point of measuremen | 12 | 48 (98.0%) | 41 (75.9%) | 89 (86.4%) | 58 (93.5%) | 30 (65.2%) | 88 (81.5%) |
| Of which Not Applicable |  | 6 (12.2%) | 8 (14.8%) | 14 (13.6%) | 15 (24.2%) | 7 (15.2%) | 22 (20.4%) |
| Participant timeline | 13 | 48 (98.0%) | 42 (77.8%) | 90 (87.4%) | 62 (100.0%) | 32 (69.6%) | 94 (87.0%) |
| Sample size: Estimated number | 14 | 43 (87.8%) | 43 (79.6%) | 86 (83.5%) | 62 (100.0%) | 45 (97.8%) | 107 (99.1%) |
| Sample size:  Outcome used for samples size calculation | 14 | 48 (98.0%) | 40 (74.1%) | 88 (85.4%) | 61 (98.4%) | 37 (80.4%) | 98 (91.7%) |
| Of which Not Applicable |  | - | - | - | 5 (8.1%) | 0 (0.0%) | 5 (4.6%) |
| Sample size: Assumed values for outcome | 14 | 23 (46.9%) | 18 (33.3%) | 41 (39.8%) | 41 (36.9%) | 20 (43.5%) | 61 (56.5%) |
| Of which Not Applicable |  | - | - | - | 5 (8.1%) | 0 (0.0%) | 5 (4.6%) |
| Sample size: Alpha value | 14 | 45 (91.8%) | 41 (75.9%) | 86 (83.5%) | 59 (95.2%) | 42 (91.3%) | 101 (93.5%) |
| Of which Not Applicable |  | - | - | - | 5 (8.1%) | 0 (0.0%) | 5 (4.6%) |
| Sample size: Statistical Power | 14 | 48 (98.0%) | 43 (79.6%) | 91 (88.4%) | 61 (98.4%) | 41 (89.1%) | 102 (94.4%) |
| Of which Not Applicable |  | - | - | - | 5 (8.1%) | 0 (0.0%) | 5 (4.6%) |
| Sample size: Rationale sample size if not derived statistically | 14 | 49 (100.0%) | 58 (100.0%) | 107 (100.0%) | 59 (95.2%) | 45 (97.8%) | 104 (96.3%) |
| Of which Not Applicable |  | 49 (100.0%) | 58 (100.0%) | 107 (100.0%) | 56 (90.3%) | 45 (97.8%) | 101 (93.5%) |
| Location of participant recruitment | 15 | 7 (14.3%) | 39 (72.2%) | 46 (44.7%) | 7 (11.3%) | 36 (78.3%) | 43 (39.8%) |
| Person(s) who will recruit participants | 15 | 5 (10.2%) | 27 (50.0%) | 32 (31.1%) | 5 (8.1%) | 31 (61.4%) | 36 (33.3%) |
| Expected recruitment rate | 15 | 7 (14.3%) | 15 (14.3%) | 22 (21.4%) | 20 (32.3%) | 25 (45.4%) | 45 (41.7%) |
| Method for generation of random sequence | 16a | 21 (42.8%) | 29 (53.7%) | 50 (48.5%) | 51 (82.3%) | 30 (65.2%) | 81 (75.0%) |
| Allocation concealment mechanism | 16b | 41 (83.7%) | 35 (64.8%) | 76 (73.8%) | 50 (80.7%) | 31 (67.4%) | 81 (75.0%) |
| Of which Not Applicable |  | - | - | - | - | - | - |
| Person who will enroll/assign participants | 16c | 16 (32.7%) | 19 (35.2%) | 35 (34.0%) | 12 (19.4%) | 17 (37.0%) | 29 (26.7%) |
| Of which Not Applicable |  | - | - | - | - | - | - |
| Blinding status of participants | 17a | 48 (98.0%) | 44 (81.%) | 92 (89.3%) | 57 (91.9%) | 32 (69.6%) | 89 (82.4%) |
| Blinding status of care providers | 17a | 48 (98.0%) | 41 (75.9%) | 89 (86.4%) | 54 (87.1%) | 38 (82.6%) | 92 (85.2%) |
| Blinding status of outcome assessors | 17a | 34 (69.4) | 32 (59.3%) | 66 (64.1%) | 44 (71.0%) | 32 (69.6%) | 76 (70.4%) |
| Conditions when unblinding is permissible | 17b | 44 (89.8%) | 34 (63.0%) | 78 (75.7%) | 58 (93.6%) | 31 (63.0%) | 87 (80.6%) |
| Of which Not Applicable |  | 16 (32.7%) | 27 (50.0%) | 43 (41.8%) | 19 (30.7%) | 24 (52.2%) | 43 (39.8%) |
| Personnel who will collect data | 18a | 23 (46.9%) | 29 (53.7%) | 52 (50.5%) | 11 (17.7%) | 24 (52.2%) | 35 (32.4%) |
| Strategies to promote participant retention and complete follow-up | 18b | 9 (18.4%) | 20 (38.0%) | 29 (28.4%) | 38 (61.3%) | 16 (34.8%) | 54 (50.0%) |
| Of which Not Applicable |  | 2 (4.1%) | 2 (3.7%) | 4 (3.9%) | - | - | - |
| Data entry and coding | 19 | 11 (22.5%) | 9 (15.5%) | 20 (18.7%) | 49 (79.0%) | 20 (43.5%) | 69 (63.9%) |
| Main analysis for primary outcome | 20a | 45 (91.8%) | 43 (79.6%) | 88 (85.4%) | 58 (93.6%) | 36 (78.3%) | 94 (87.0%) |
| Definition of subgroup categories | 20b | 44 (89.8%) | 50 (92.6%) | 94 (91.3%) | 45 (72.6%) | 39 (84.8%) | 84 (87.8%) |
| Of which Not Applicable |  | 31 (63.3%) | 57 (87.0%) | 78 (75.7%) | 28 (45.2%) | 29 (63.0%) | 57 (52.8%) |
| Definition of analysis population | 20c | 43 (87.7%) | 31 (57.4%) | 74 (71.8%) | 57 (91.9%) | 17 (37.0%) | 74 (68.5%) |
| DMC is planned or why it is not planned | 21a | 35 (71.4%) | 20 (37.0%) | 55 (53.4%) | 41 (66.1%) | 22 (47.8%) | 63 (58.3%) |
| Who has authority to stop the trial | 21b | 40 (81.6%) | 19 (35.2%) | 59 (57.3%) | 47 (75.8%) | 20 (43.5%) | 67 (62.0%) |
| Anticipated/unanticipated adverse events collection | 22 | 49 (100.0%) | 36 (66.7%) | 85 (82.5%) | 62 (100.0%) | 39 (84.8%) | 101 (93.5%) |
| Audits/external monitoring described | 23 | 10 (20.4%) | 4 (7.4%) | 14 (13.6%) | 26 (41.9%) | 3 (5.5%) | 29 (26.9%) |
| Of which Not Applicable |  | - | - | - | 0 (0.0%) | 1 (2.2%) | 1 (0.93%) |
| Research ethics approval | 24 | 49 (100.0%) | 54 (100.0%) | 103 (100.0%) | 62 (100.0%) | 46 (100.0%) | 108 (100.0%) |
| Process for making amendments described | 25 | 27 (55.1%) | 8 (14.8%) | 35 (34.0%) | 50 (80.7%) | 21 (45.7%) | 71 (65.7%) |
| Informed Consent process described | 26a | 45 (91.8%) | 46 (84.2%) | 91 (88.4%) | 53 (85.5%) | 35 (75.1%) | 88 (81.5%) |
| Process to obtain additional consent for collection and use of data and biological specimens | 26b | 45 (91.2%) | 52 (96.3%) | 97 (94.2%) | 49 (79.0% | 45 (97.8) | 94 (87.0%) |
| Of which Not Applicable |  | 34 (69.4%) | 45 (83.3%) | 79 (76.7%) | 42 (91.3%) | 39 (62.9%) | 81 (75.0%) |
| Confidentiality of data | 27 | 43 (87.8%) | 37 (68.5%) | 80 (77.7%) | 48 (77.4%) | 34 (73.9%) | 82 (75.9%) |
| Declaration of Interests | 28 | 8 (16.3%) | 4 (7.4%) | 12 (11.7%) | 60 (96.8%) | 10 (21.7%) | 70 (64.8%) |
| Who will have access to full dataset | 29 | 4 (8.2%) | 16 (29.6%) | 20 (19.4%) | 18 (29.0%) | 7 (15.2%) | 25 (23.2%) |
| Ancillary and post-trial care | 30 | 4 (8.2%) | 8 (14.8%) | 12 (11.7%) | 29 (46.8%) | 31 (67.4%) | 60 (55.6%) |
| Plans to disseminate trial results to key stakeholders/publication provided | 31a | 34 (69.4%) | 39 (72.2%) | 73 (70.9%) | 28 (45.2%) | 32 (69.6%) | 60 (55.6%) |
| Authorship eligibility criteria | 31b | 10 (20.4%) | 16 (29.6%) | 26 (25.2%) | 23 (37.1%) | 15 (32.6%) | 38 (35.2%) |
| Plans for granting access to full trial protocol | 31c | 0 (0.0%) | 1 (1.9%) | 1 (1.0%) | 6 (9.7%) | 2 (4.4%) | 8 (7.4%) |
| Consent forms provided | 32 | 49 (100.0%) | 58 (100.0%) | 107 (100.0%) | 62 (100.0%) | 46 (100.0%) | 108 (100.0%) |
| Details of specimen collection | 33 | 49 (100.0%) | 53 (92.2%) | 102 (99.0%) | 55 (88.7%) | 42 (91.3%) | 97 (89.8%) |
| Of which Not Applicable |  | 34 (69.4%) | 46 (85.2%) | 80 (77.7%) | 10 (16.1%) | 31 (67.4%) | 41 (38.0%) |

**Table S3:** Absolute increase in adequate reported selected SPIRIT item components (2012 vs 2016) for components that were not commonly reported (i.e. ≤50%) in 2012.

|  |  | **2012** | | | **2016** | | | **2012 vs 2016** |
| --- | --- | --- | --- | --- | --- | --- | --- | --- |
| **Variable** | **Spirit Item Number** | **Industry sponsorship (n=49)** | **Non-industry sponsorship (n=54)** | **Total 2012 (n=103)** | **Industry sponsorship (n=62)** | **Non-industry sponsorship (n=46)** | **Total 2016 (n=108)** | **Absolute increase** |
| Basic study design in Title | 1 | 31 (63.3%) | 9 (16.7%) | 40 (38.8%) | 53 (85.5%) | 24 (52.2%) | 77 (71.3%) | 32.5% |
| Trial registration | 2 | 33 (67.4%) | 17 (31.5%) | 50 (48.5%) | 54 (87.1%) | 19 (41.3%) | 73 (67.6%) | 22.1% |
| Names of protocol contributors/ authors | 5a | 4 (8.2%) | 8 (13.8%) | 12 (11.2%) | 5 (8.1%) | 7 (15.2%) | 12 (11.1%) | -0.1% |
| Name and contact details of sponsor | 5b | 12 (24.5%) | 16 (29.6%) | 28 (27.2%) | 31 (50.0%) | 31 (67.4%) | 62 (57.4%) | 30.2% |
| Research question described and justified | 6a | 11 (22.5%) | 9 (16.7%) | 20 (19.4%) | 35 (56.5%) | 13 (28.3%) | 48 (44.4%) | 25.0% |
| Eligibility criteria for study centres and who will perform the intervention | 10 | 9 (18.4%) | 24 (44.4%) | 33 (32.0%) | 8 (12.9%) | 24 (52.2%) | 32 (29.6%) | -2.4% |
| Sample size: Assumed values for outcome | 14 | 23 (46.9%) | 18 (33.3%) | 41 (39.8%) | 41 (36.9%) | 20 (43.5%) | 61 (56.5%) | 16.7% |
| Location of participant recruitment | 15 | 7 (14.3%) | 39 (72.2%) | 46 (44.7%) | 7 (11.3%) | 36 (78.3%) | 43 (39.8%) | -4.9% |
| Person(s) who will recruit participants | 15 | 5 (10.2%) | 27 (50.0%) | 32 (31.1%) | 5 (8.1%) | 31 (61.4%) | 36 (33.3%) | 2.2% |
| Expected recruitment rate | 15 | 7 (14.3%) | 15 (14.3%) | 22 (21.4%) | 20 (32.3%) | 25 (45.4%) | 45 (41.7%) | 20.3% |
| Method for generation of random sequence | 16a | 21 (42.8%) | 29 (53.7%) | 50 (48.5%) | 51 (82.3%) | 30 (65.2%) | 81 (75.0%) | 26.5% |
| Person who will enroll/assign participants | 16c | 16 (32.7%) | 19 (35.2%) | 35 (34.0%) | 12 (19.4%) | 17 (37.0%) | 29 (26.7%) | -7.3% |
| Strategies to promote participant retention and complete follow-up | 18b | 9 (18.4%) | 20 (38.0%) | 29 (28.4%) | 38 (61.3%) | 16 (34.8%) | 54 (50.0%) | 21.6% |
| Data entry and coding | 19 | 11 (22.5%) | 9 (15.5%) | 20 (18.7%) | 49 (79.0%) | 20 (43.5%) | 69 (63.9%) | 45.2% |
| Audits/external monitoring described | 23 | 10 (20.4%) | 4 (7.4%) | 14 (13.6%) | 26 (41.9%) | 3 (5.5%) | 29 (26.9%) | 13.3% |
| Process for making amendments described | 25 | 27 (55.1%) | 8 (14.8%) | 35 (34.0%) | 50 (80.7%) | 21 (45.7%) | 71 (65.7%) | 31.7% |
| Declaration of Interests | 28 | 8 (16.3%) | 4 (7.4%) | 12 (11.7%) | 60 (96.8%) | 10 (21.7%) | 70 (64.8%) | 53.1% |
| Who will have access to full dataset | 29 | 4 (8.2%) | 16 (29.6%) | 20 (19.4%) | 18 (29.0%) | 7 (15.2%) | 25 (23.2%) | 3.8% |
| Ancillary and post-trial care | 30 | 4 (8.2%) | 8 (14.8%) | 12 (11.7%) | 29 (46.8%) | 31 (67.4%) | 60 (55.6%) | 43.9% |
| Authorship eligibility criteria | 31b | 10 (20.4%) | 16 (29.6%) | 26 (25.2%) | 23 (37.1%) | 15 (32.6%) | 38 (35.2%) | 10.0% |
| Plans for granting access to full trial protocol | 31c | 0 (0.0%) | 1 (1.9%) | 1 (1.0%) | 6 (9.7%) | 2 (4.4%) | 8 (7.4%) | 6.4% |

**Table S4**: Results from beta regression to assess what characteristics are associated with higher proportion of adequate reporting.

| **Independent variable** | **Coefficient** | **95% Confidence interval** | **p-value** |
| --- | --- | --- | --- |
| Year of approval (2012 vs 2016) | 0.082 | 0.052-0.113 | <0.001 |
| Sponsor (industry vs non-industry) | 0.286 | 0.159-0.413 | <0.001 |
| Planned sample size^a^ | -0.017 | -0.060-0.025 | 0.420 |
| Multicentre (vs single centre) | 0.399 | 0.253-0.545 | <0.001 |

^a^In increments of 1000

**References**

1. Gryaznov D, Odutayo A, von Niederhausern B, Speich B, Kasenda B, Ojeda-Ruiz E, et al. Rationale and design of repeated cross-sectional studies to evaluate the reporting quality of trial protocols: the Adherence to SPIrit REcommendations (ASPIRE) study and associated projects. Trials. 2020;21(1):896.
